# The impact of perioperative red blood cell transfusion on the prognosis of colorectal cancer

**DOI:** 10.3389/fsurg.2022.927787

**Published:** 2022-07-21

**Authors:** Ting Jiang, Kun Liu, Zexin Chen

**Affiliations:** ^1^ Department of Blood Transfusion, Chongqing Emergency Medical Center, Chongqing University Central Hospital, Chongqing, China; ^2^ Key Laboratory of Clinical Laboratory Dignostics (Ministry of Education), College of Laboratory Medicine, Chongqing Medical University, Chongqing, China

**Keywords:** perioperative, red blood cell transfusion, prognosis, colorectal cancer, severity

## Abstract

**Background:**

There is no consensus on the effect of red blood cell transfusion on colorectal cancer (CRC). This study examined the impact of perioperative red blood cell transfusion on postoperative complications, recurrence, and mortality in patients with CRC.

**Methods:**

In this retrospective cohort study, 219 CRC patients admitted to Chongqing Emergency Medical Center, and Chongqing University Central Hospital from 2008 to 2019 were divided into transfusion (*n* = 75) and non-transfusion (*n* = 144) groups. Univariate and multivariate Logistic regression analysis were used to analyze the effects of blood transfusion on the severity of postoperative complications in patients with CRC, and univariate and multivariate Cox regression was performed to analyze the effects of blood transfusion on postoperative death and recurrence.

**Results:**

Twenty-two (29.33%) patients in the transfusion group were intermediate or advanced severity of postoperative complications, 31 (41.33%) patients died in the transfusion group, and 55 (73.33%) patients occurred recurrence of the CRC, with the median follow-up time being 24.57(14.50,36.37) months. Our result showed that perioperative red blood cell transfusion was associated with an increased risk of intermediate or advanced severity of postoperative complications in CRC patients [odds ratio (OR) = 3.368, 95% CI, 1.146–9.901]. And perioperative red blood cell transfusion increased the risk of postoperative death [hazard ratio (HR) = 2.747, 95% CI, 1.048–7.205] and recurrence in patients with CRC (HR = 2.168, 95% CI, 1.192–3.943).

**Conclusion:**

Our finding demonstrated that perioperative red blood cell transfusion was associated with severity of complications, recurrence, and death in CRC patients. However, further studies are still needed to confirm the adverse effects of red blood cell transfusions in CRC patients.

## Introduction

Colorectal cancer (CRC) is a malignant tumor of the digestive tract, ranking the 3rd most common cancer in males and the 2nd most common cancer in females (1, 2). CRC is also the 4th most common cause of cancer-related death worldwide, with 1.8 million new cases are diagnosed annually worldwide, about 900,000 individuals die from this malignancy (1, 3). It is reported that about 50% of patients with CRC will have recurrence and distant metastasis after radical resection (4). The 5-year survival rate of patients with early colorectal cancer is ideal, at about 90%, while the 5-year survival rate of patients with recurrence and metastasis of colorectal cancer is less than 12% (5). It is critical to quarry the factors that may affect the prognosis of CRC to improve the survival of CRC patients.

Patients with CRC often require blood transfusions due to the presence of iron deficiency anemia or chronic blood loss (6). Perioperative transfusion rates in CRC patients range from 10% to 68% (7). Previous studies have suggested a link between transfusion and worse cancer outcomes (8, 9), whereas others found transfusion was not associated with the prognosis of cancer patients (10), or argue that there may be a link, but that it is likely to be noncausally related to blood transfusion (11). Red blood cell transfusion is an important aspect of blood component therapy (12). Red blood cell transfusion can improve tissue oxygenation and can be used to treat hospitalized patients with acute and chronic anemia. The use of red blood cell transfusion during surgery has also saved countless lives (12, 13). However, there is little consensus exists about the actual effects of red blood cell transfusions in general, and even less so in CRC as a separate entity. Given the conflicting result between perioperative blood transfusion and oncologic outcomes and limited studies on red blood cell transfusions, this study was performed.

Herein, current study was to examine the impact of perioperative red blood cell transfusion on severity of postoperative complications, recurrence, and death in CRC patients. This study may provide a valuable clinical reference for perioperative red blood cell transfusion in CRC patients and prognosis improvement.

## Methods

### Design and study population

In this retrospective cohort study, 241 patients at Chongqing Emergency Medical Center, and Chongqing University Central Hospital who underwent CRC resection were collected between 2008 and 2019. The inclusion criteria were presented as: (1) age ≥18 years old; (2) colonoscopy and histopathologic biopsy confirmed CRC; (3) elective surgical treatment; (4) The laboratory, pathological examination, and outcome data were complete. The exclusion criteria were as follows: (1) patients with distant metastasis or recurrent cancer; (2) death during the perioperative period; (3) patients with other metabolic diseases such as diabetes, hyperthyroidism, hypothyroidism, etc.; (4) patients complicated with other tumors; (5) patients with coagulation dysfunction or other blood system diseases; (6) patients who had taken other investigational drugs or was participating in other clinical studies within 1 month prior to study inclusion. Finally, 219 CRC patients were included, 75 received a blood transfusion during the perioperative period, which was called the transfusion group. The 144 patients who did not receive a blood transfusion during perioperative period were referred to as the non-transfusion group.

### Data collection

Data were collected, including (1) demographics: age (year), gender; (2) disease status: tumor site, tumor size (cm^3^), tumor differentiation, the American Society of Anesthesiologists (ASA) classification, tumor node metastasis (TNM) classification; (3) surgical situation: surgical methods, operation time (min), intraoperative blood loss (ml); preoperative adjuvant radiotherapy and chemotherapy; postoperative adjuvant radiotherapy and chemotherapy; (4) laboratory events: hemoglobin, albumin; (5) follow-up period (month).

### Variables and outcome measures

The perioperative period refers to the duration from when the patient decides to receive surgery until the basic recovery period after surgery; this includes the time before, during, and after surgery.

#### The severity of surgical complications

The severity of complications was assessed by the Clavien-Dindo classification system was used to determine. Grade 1 included minor risk events that do not require treatment (analgesic, antipyretic, antiemetic, and antidiarrheal drugs or drugs required for lower urinary tract infection were except). Grade 2 complications referred to the potentially life-threatening complications that required intervention or more than twice the median length of hospital stay. Grade 2 was divided into 2 subgroups according to the aggressiveness of the complications chosen for treatment; grade 2a complications required only medications and grade 2b required an invasive procedure. Grade 3 indicated the complications resulting in permanent disability or organ resection. Grade 4 complication were defined as the death of a patient due to a complication (14). In this study, grade 1 was as low severity of complication, grades 2 and above were as intermediate and advanced severity.

#### Tumor recurrence

Tumor recurrence was defined as any radiologic or histologic evidence of tumor growth in the previous surgical field or distant organs (15).

### Statistical analysis

Continuous variables with normal distribution were expressed as mean ± standard deviation (Mean ± SD), and t-test was used for comparison between groups. Skewed distribution was exhibited as [M (Q1, Q3)], and Wilcoxon rank sum test was used for comparison between groups. The categorical variables were described by *N* (%), the ordered categorical variables were tested by the Wilcoxon rank sum test, and the disordered categorical variables were tested by chi-square test. Univariate and multivariate Logistic regression were used to analyze the effects of blood transfusion on postoperative complication severity in patients with CRC, and univariate and multivariate Cox regression was applied for analyzing the effects of blood transfusion on postoperative death and recurrence. All statistical tests were two-tailed test. *P *< 0.05 was considered to be statistically significant. Using SAS 9.4 software (SAS Institute, Inc, Cary, North Carolina) for the statistical analysis.

## Results

### Comparison of clinical characteristics of included study subjects

**Figure 1:**
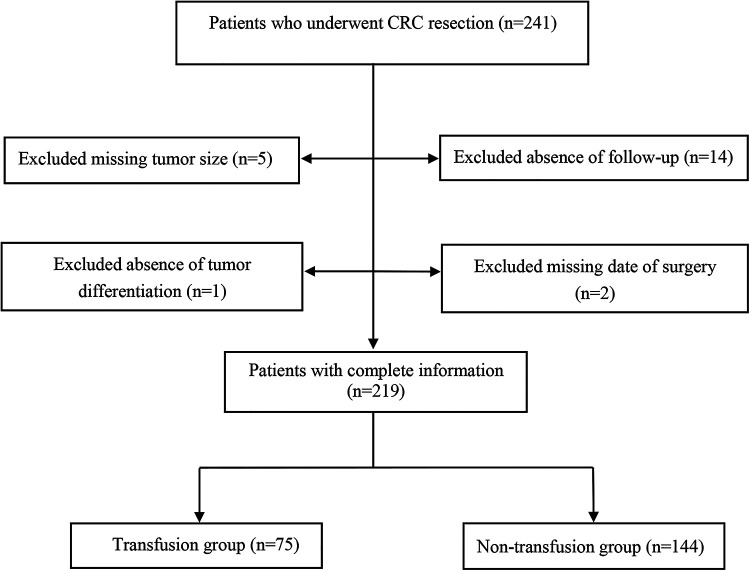
The flow chart of data collection.

Among the 219 enrolled patients, 75 (34.25%) were in the transfusion group and 144 (65.75%) were in the non-transfusion group. The median follow-up time of the 219 patients was 24.57 months. The mean age of the transfusion group was higher than that of the non-transfusion group (*t* = −3.970, *P *< 0.001). The distribution of tumor sites was different between the transfusion group and the non-transfusion group (*χ*^2 ^= 15.356, *P *< 0.001). Tumors in the transfusion group were larger than those in the non-transfusion group (Z = 5.130, *P *< 0.001). The degree of tumor differentiation in the transfused group was higher than that in the non-transfused group (Z = 3.202, *P *= 0.001). The amount of intraoperative blood loss in the transfusion group was higher than that in the non-transfusion group (Z = 5.101, *P *< 0.001). The hemoglobin of the transfusion group was lower than that of the non-transfusion group (*t* = 11.350, *P *< 0.001). Albumin in the transfusion group was lower than that in the non-transfusion group (*t* = 6.720, *P *< 0.001) (Table 1). An overview of this study and the data collection selection are described in Figure 1. The survival curves estimated by different ages, tumor sizes, TNM stage, hemoglobin levels, and albumin levels were plotted in Supplementary Figure S1–S5.

**Table 1:** Baseline characteristics of the transfusion and non-transfusion groups.

Variables	Total	Non-transfusion	Transfusion	*t/χ^2^/Z* value	*P-*value
Total group, *n* (%)	219	144 (65.75)	75 (34.25)		
Age (year), Mean ± SD	64.61 ± 14.09	61.97 ± 14.05	69.68 ± 12.79	−3.970	<0.001
Gender, *n* (%)				3.574	0.059
Male	130 (59.36)	92 (63.89)	38 (50.67)		
Female	89 (40.64)	52 (36.11)	37 (49.33)		
Tumor sites, *n* (%)				15.356	<0.001
Left colon	66 (30.14)	46 (31.94)	20 (26.67)		
Right colon	80 (36.53)	40 (27.78)	40 (53.33)		
Rectum	73 (33.33)	58 (40.28)	15 (20.00)		
Tumor size (cm^3^), M (Q_1_,Q_3_)	48.00 (18.00,100.00)	36.00 (13.50,80.00)	80.00 (40.00,247.50)	5.130	<0.001
Tumor differentiation, *n* (%)				3.202	0.001
Well	20 (9.13)	17 (11.81)	3 (4.00)		
Moderate	176 (80.37)	118 (81.94)	58 (77.33)		
Poor/undifferentiated	23 (10.50)	9 (6.25)	14 (18.67)		
ASA classification, *n* (%)				−0.218	0.828
Stage I	102 (46.58)	64 (44.44)	38 (50.67)		
Stage II	102 (46.58)	74 (51.39)	28 (37.33)		
Stage III	15 (6.85)	6 (4.17)	9 (12.00)		
TNM classification, *n* (%)				2.105	0.037
0 and I	37 (16.89)	31 (21.53)	6 (8.00)		
II	88 (40.18)	57 (39.58)	31 (41.33)		
III	90 (41.10)	52 (36.11)	38 (50.67)		
IV	4 (1.83)	4 (2.78)	0 (0.00)		
Surgical situation, *n* (%)				1.759	0.185
Laparotomy	86 (39.27)	52 (36.11)	34 (45.33)		
Peritoneoscope	133 (60.73)	92 (63.89)	41 (54.67)		
Operation time (min), *n* (%)				0.166	0.684
≤150	23 (10.50)	16 (11.11)	7 (9.33)		
>150	196 (89.50)	128 (88.89)	68 (90.67)		
Intraoperative blood loss (ml), *n* (%)				5.101	<0.001
<500	201 (91.78)	142 (98.61)	59 (78.67)		
501–1000	14 (6.39)	2 (1.39)	12 (16.00)		
>1000	4 (1.83)	0 (0.00)	4 (5.33)		
Hemoglobin (g/L), Mean ± SD	117.90 ± 24.43	128.63 ± 18.50	97.29 ± 21.02	11.350	<0.001
Albumin (g/L), Mean ± SD	38.90 ± 5.44	40.65 ± 4.35	35.56 ± 5.76	6.720	<0.001
Preoperative adjuvant radiotherapy and chemotherapy, *n* (%)				–	0.667
No	213 (97.26)	139 (96.53)	74 (98.67)		
Yes	6 (2.74)	5 (3.47)	1 (1.33)		
Postoperative adjuvant radiotherapy and chemotherapy, *n* (%)				χ^2 ^= 0.006	0.938
No	15 (6.85)	10 (6.94)	5 (6.67)		
Yes	204 (93.15)	134 (93.06)	70 (93.33)		
Perioperative blood transfusion (ml), *n* (%)					
<800			64 (85.33%)		
≥800			11 (14.67%)		
Follow-up time (months)	24.57 (14.50,36.37)				

*Notes: ASA, American Society of Anesthesiologists; TNM, tumor node metastasis.*

### Prognosis analysis of transfusion group and non-transfusion group

The severity of postoperative complications in 22 (29.33%) patients in the transfusion group and 16 (11.11%) patients in the non-transfusion group were intermediate or advanced. The proportion of intermediate or advanced complications in the transfusion group was higher than that in the non-transfusion group (*χ*^2 ^= 11.416, *P *= 0.001). Thirty-one (41.33%) patients in the transfusion group died during the follow-up period, while 11 (7.64%) patients in the non-transfusion group died during the follow-up period. The mortality rate in the transfusion group was higher than that in the non-transfusion group (*χ*^2 ^= 36.121, *P *< 0.001). Fifty-five (73.33%) patients in the transfusion group relapsed during the follow-up period, and 34 (23.61%) patients in the non-transfusion group relapsed during the follow-up period. The recurrence rate in the transfusion group was higher than that in the non-transfusion group (*χ*^2 ^= 50.540, *P *< 0.001) (Table 2).

**Table 2:** Analysis of prognosis of blood transfusion group and non-transfusion group.

Variables	Total	Non-transfusion	Transfusion	*χ*^2^ value	*P*
Postoperative complications, *n* (%)	219			11.416	0.001
Inferiority	181 (82.65)	128 (88.89)	53 (70.67)		
Intermediate or advances	38 (17.35)	16 (11.11)	22 (29.33)		
Mortality, *n* (%)	42 (19.18)	11 (7.64)	31 (41.33)	36.121	<0.001
Recurrence, *n* (%)	89 (40.64)	34 (23.61)	55 (73.33)	50.540	<0.001

### Effects of blood transfusion on postoperative complications in patients with CRC

**Figure 2:**
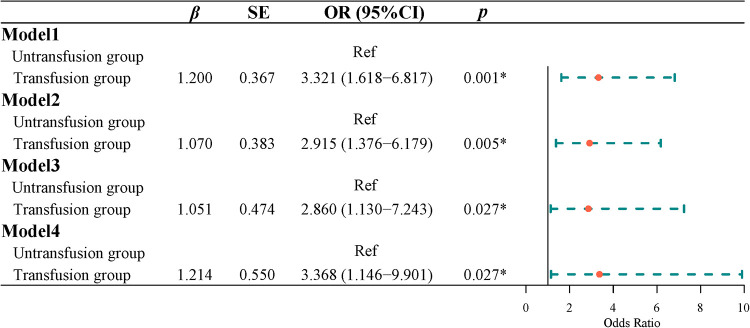
The effect of blood transfusion on postoperative complications.

Univariate Logistic regression results showed that the risk of postoperative complications with intermediate or higher severity in the transfusion group was 3.321 times higher than that in the non-transfusion group [odds ratio (OR) = 3.321, 95% CI, 1.618–6.817]. Multivariate Logistic regression results indicated that after adjusting for age and gender, the risk of postoperative complications of intermediate or higher severity in the transfusion group was 2.915 times higher than that in the non-transfusion group (OR = 2.915, 95% CI, 1.376–6.179). After adjusting for age, sex, tumor site, tumor size, tumor differentiation, ASA classification, and TNM classification, the risk of a medium-high risk of postoperative complication severity in the transfusion group was 2.860 times higher than that in the non-transfusion group (OR = 2.860, 95% CI, 1.130–7.243). After adjusting for age, sex, tumor site, tumor size, tumor differentiation, ASA classification, TNM stage, hemoglobin, and albumin, the risk of the severity of postoperative complications in the transfusion group was 3.368 times higher than that in the non-transfusion group (OR = 3.368, 95% CI, 1.146–9.901) (Figure 2).

### Effect of blood transfusion on mortality in patients with CRC

**Figure 3:**
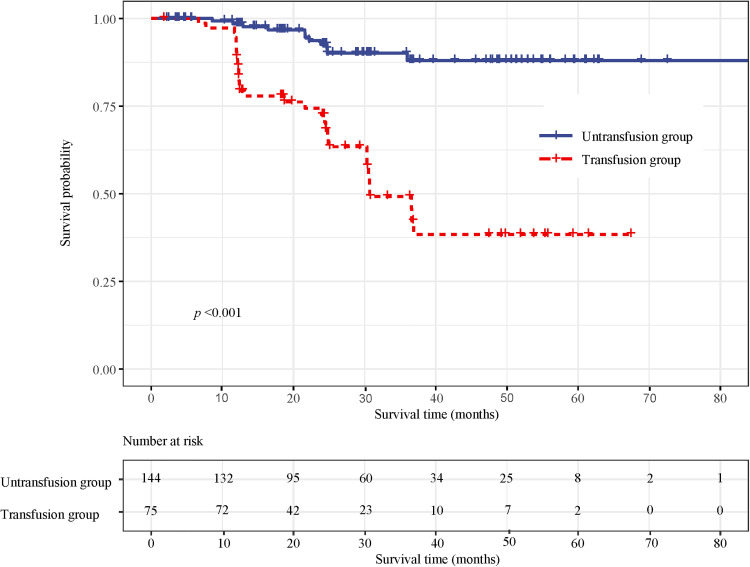
Survival curves of death between the transfused and non-transfused group.

**Figure 4:**
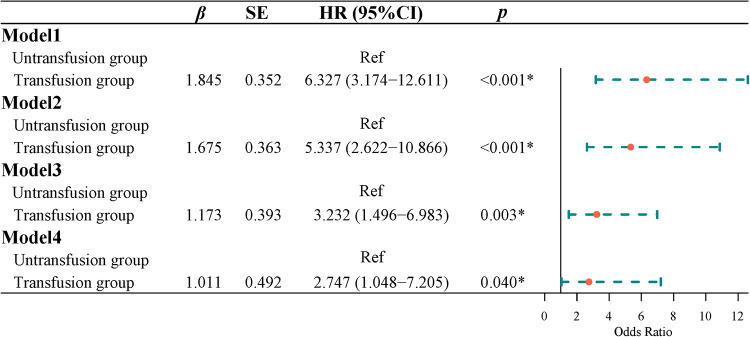
The effect of blood transfusion on postoperative death.

Survival curve results showed that the survival time of the transfusion group was shorter than that of the non-transfusion group (log-rank test, *P *< 0.001) (Figure 3). Cox regression was used to analyze the effect of blood transfusion on postoperative mortality of patients with CRC. Univariate Cox regression showed that the risk of postoperative death in the transfusion group was 6.327-fold higher than that in the non-transfusion group (HR = 6.327, 95% CI, 3.174–12.611). After adjusting for age and sex, multivariate cox regression results showed that the risk of postoperative death in the transfusion group was 5.337 times higher than that in the non-transfusion group (HR = 5.337, 95% CI, 2.622–10.866). After adjusting for age, sex, tumor site, tumor size, tumor differentiation, ASA classification, and TNM stage, the risk of postoperative death in the transfusion group was 3.232-fold higher than that in the non-transfusion group (HR = 3.232, 95% CI, 1.496–6.983). After adjusting for age, sex, tumor site, tumor size, tumor differentiation, ASA classification, TNM stage, hemoglobin, and albumin, the risk of postoperative death in the transfusion group was 2.747 times higher than that in the non-transfusion group (HR = 2.747, 95% CI, 1.048–7.205) (Figure 4).

### Effect of blood transfusion on recurrence in patients with CRC

**Figure 5:**
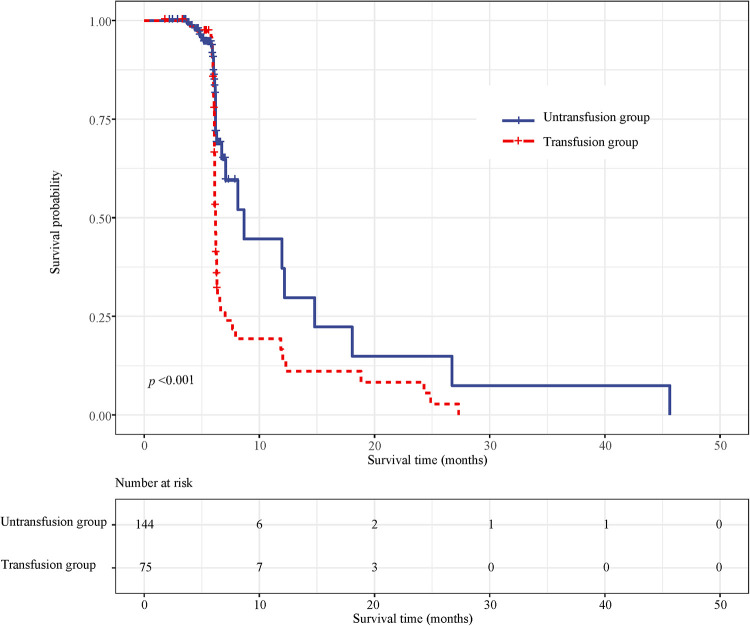
Survival curves of the recurrence between the transfused and non-transfused groups.

**Figure 6:**
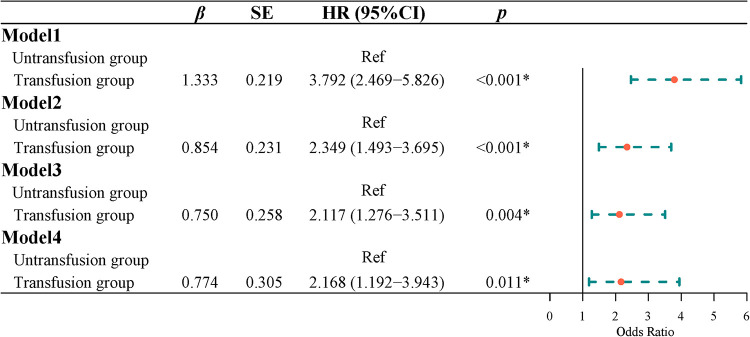
The effect of blood transfusion on postoperative recurrence.

Survival curve results demonstrated that the survival time of the transfusion group was shorter than that of the non-transfusion group (log-rank test, *P *< 0.001) (Figure 5). COX regression was used to analyze the effect of blood transfusion on the postoperative recurrence of colorectal cancer patients. The univariate COX regression results showed that the risk of recurrence in the blood transfusion group was 3.792 times that of the non-transfusion group (HR = 3.792, 95% CI, 2.469–5.826). Multivariate COX regression results showed that after controlling for age and gender, the risk of recurrence after the blood transfusion group was 2.349 times that of the non-transfusion group (HR = 2.349, 95% CI, 1.493–3.695). After adjusting for age, sex, tumor location, tumor size, and tumor differentiation, the risk of recurrence in the blood transfusion group was 2.178 times that of the non-transfusion group (HR = 2.178, 95% CI, 1.351–3.511). After adjusting for age, gender, tumor location, tumor size, tumor differentiation, ASA classification, and TNM staging, the risk of postoperative recurrence in the blood transfusion group was 2.117 times that of the non-transfusion group (HR = 2.117, 95% CI, 1.276–3.511). After adjusting for age, gender, tumor location, tumor size, tumor differentiation, ASA classification, TNM staging, hemoglobin, and albumin, the risk of recurrence in the blood transfusion group was 2.168 times that of the non-transfusion group (HR = 2.168, 95% CI, 1.192–3.943) (Figure 6).

## Discussion

Few studies evaluating the effect of red blood cell transfusion on CRC (9). This study was conducted to examine whether perioperative red blood cell transfusions is associated with poor outcomes in CRC patients. Our study indicated perioperative red blood cell transfusion had negative impacts on the complications, recurrence, and mortality in patients with CRC. Perioperative red blood cell transfusion increased intermediate or higher severity of complications and the risk of postoperative death and recurrence in patients with CRC. The effect of transfusions on clinical outcomes was similar in studies published in earlier (2000–2009) and (2010–2020) decades, which supports the repeatability and consistency of our results.

The result of this study showed that perioperative red blood cell transfusion increased the risk of postoperative death in CRC patients. A previous study (9) showed a significantly higher mortality rate in CRC patients with perioperative packed red blood cell transfusions. Similarly, Qiu et al. (16) found that perioperative blood transfusion increased postoperative mortality in CRC patients. A meta-analysis (17) suggested that in patients with CRC undergoing surgery, allogeneic red blood cell transfusions are associated with an increased mortality. It is hypothesized that the detrimental effect of blood transfusion on cancer outcomes results from immunological derangements, including changes in circulating lymphocytes, helper T-cell, suppressor T-cell ratios, and B-cell function (18). Results of a propensity score analysis of 4,030 patients showed patients with the larger volume of perioperative transfusions were associated with a greater chance of mortality (19). We speculate that the effect of transfusion volume may be related to surgical complexity and aggressiveness of cancer, reflecting perioperative course and disease severity rather than the amount of transfusion *per se* (20). Further research concerning the effect of red blood cell transfusions volume on prognosis may also be necessary.

A study (16) by Qiu et al. found that the local recurrence rate of transfused patients was significantly higher than those of non-transfused patients, which is consistent with our results. During the perioperative period, global inflammation results in a reduced state of host tumor immunosurveillance, increasing the risk of recurrence of micrometastases that expand or fail to eradicate the minimal residual disease (21, 22). In addition, intraoperative tumor management increases the load of circulating malignant cells, which may lead to long-distance spread. Transfusion further exacerbates this pro-tumor environment (23). Nevertheless, the impact of perioperative red blood cell transfusion on the risk of cancer recurrence needed further evaluation.

Our study demonstrated that perioperative red blood cell transfusion increased the severity of postoperative complications. This finding is supported by observations reported by other authors (24–26). Tamini et al. (26) found that blood transfusion is associated with severe postoperative complications following colectomy in CRC patients. Study (25) by Papageorge et al. indicated that preoperative transfusion was an independent predictor of complications in CRC patients with mild and moderate anemia.

Our findings provide additional insights into the relationship between blood transfusion and postoperative tumor outcomes in CRC patients. This article reminds us of the importance of proper blood management for patients with CRC. Actively entangle the anemia of patients with CRC, minimize the loss of blood during the perioperative period, take other favorable measures to increase hemoglobin levels, reduce the need for perioperative blood transfusion, and improve the clinical prognosis of patients with CRC.

The current study has several limitations. First, given the retrospective and non-randomized small sample design, we cannot ascertain causation but merely an association between perioperative red blood cell transfusion and prognosis of CRC. Second, because of the different sources of blood, it might be possible that this causes a selection bias. Further research should explore the discrepancy. Furthermore, conclusions about tumor recurrence need to be interpreted with caution because of the short follow-up period. Finally, potential confounding factors may affect our results. Future prospective studies with long follow-up times and large sample sizes are needed to demonstrate or disprove the effect of red blood cell transfusion on the outcomes in CRC patients.

## Conclusion

In our study, the use of perioperative red blood cell transfusion was associated with postoperative complications, recurrence, and mortality in patients with CRC. Our results may serve as an important clinical reflection of blood transfusion for CRC patients. However, further studies are needed to explain the adverse effects of red blood cell transfusions in CRC patients.

## Data Availability

The raw data supporting the conclusions of this article will be made available by the authors, without undue reservation.
