# Supplementary figures and images for: The impact of perioperative red blood cell transfusion on the prognosis of colorectal cancer
Source: Front Surg. 2022 Jul 21;9:927787. doi: 10.3389/fsurg.2022.927787 (PMC9349360; doi:10.3389/fsurg.2022.927787)

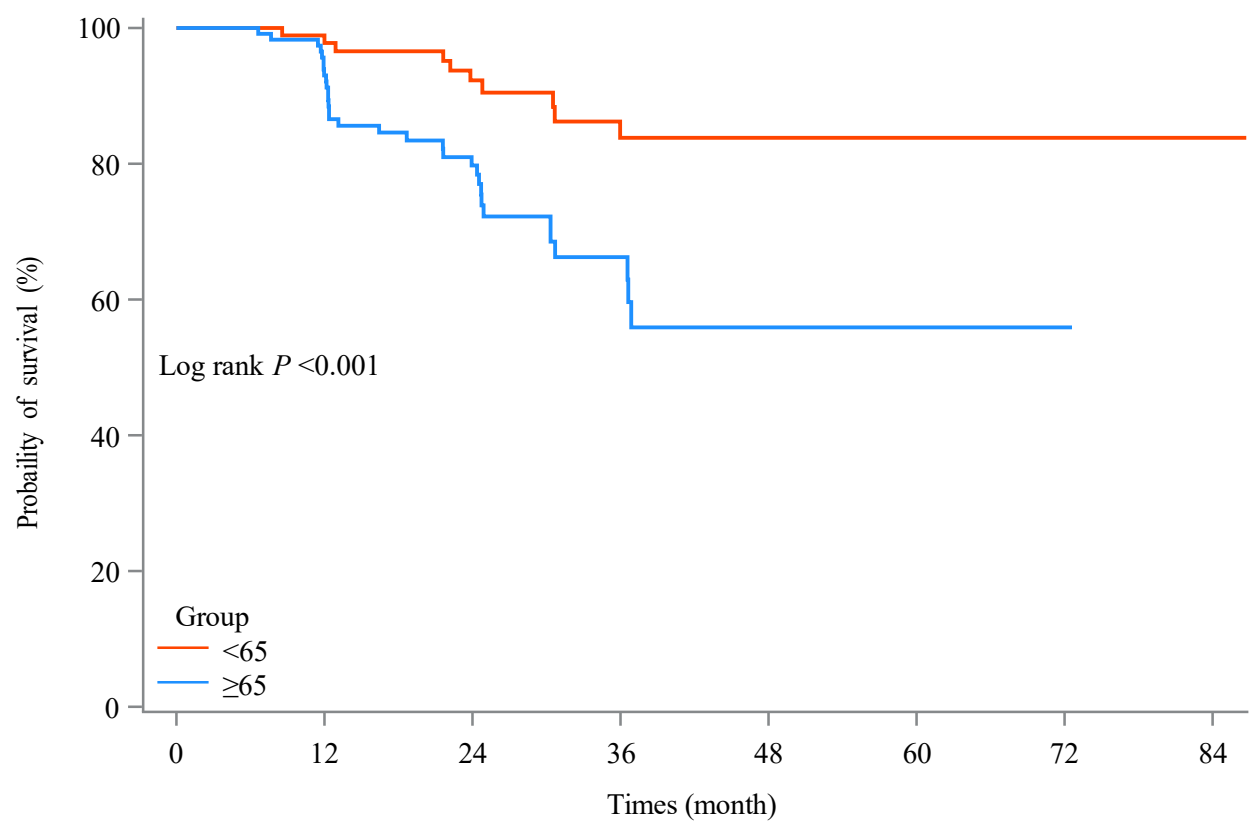

| No. at risk |     |     |    |    |    |   |   |   |
|-------------|-----|-----|----|----|----|---|---|---|
| <65         | 103 | 89  | 64 | 35 | 28 | 7 | 1 | 1 |
| ≥65         | 116 | 106 | 65 | 22 | 11 | 3 | 1 | 0 |

Supplement: Supplementary file 1 [file Data_Sheet_2_v1.pdf]

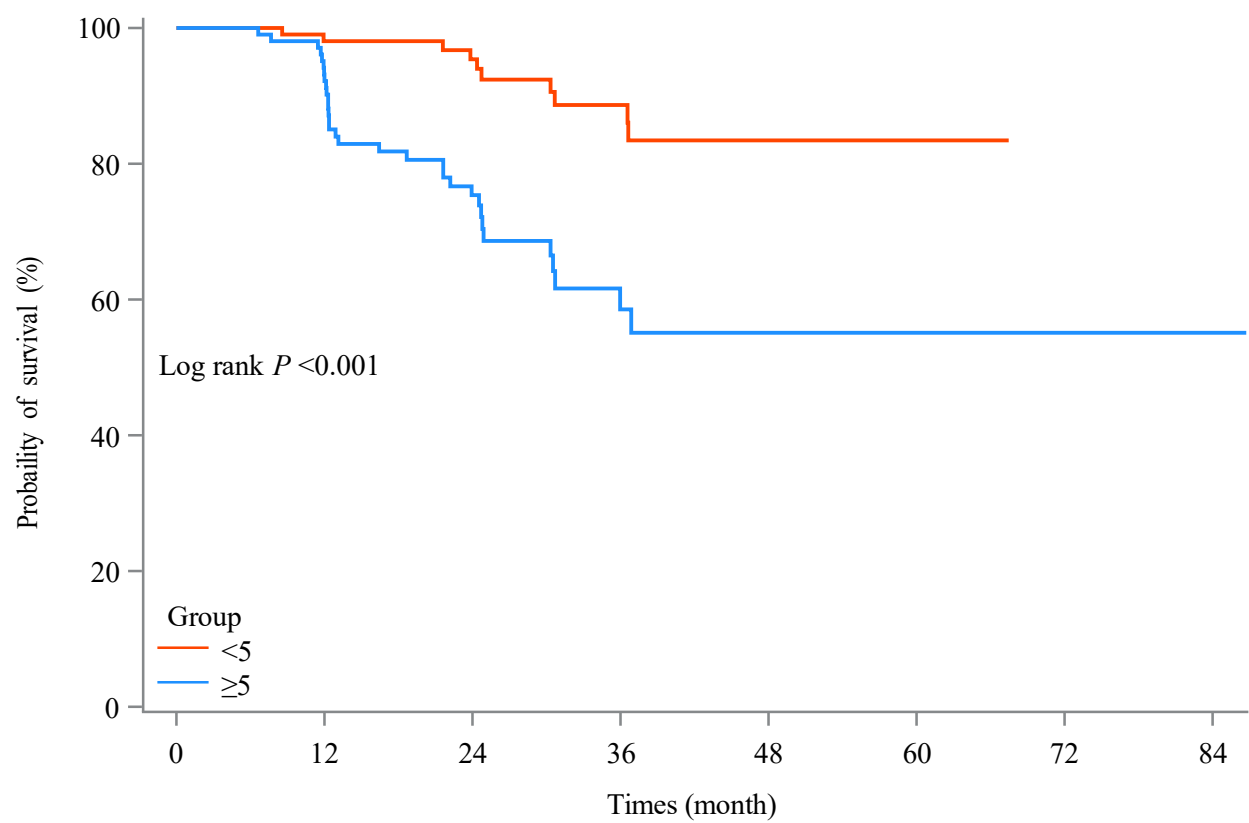

| No. at risk |     |     |    |    |    |   |   |   |
|-------------|-----|-----|----|----|----|---|---|---|
| $<5$        | 109 | 100 | 71 | 38 | 26 | 6 | 0 |   |
| $\geq 5$    | 110 | 95  | 58 | 19 | 13 | 4 | 2 | 1 |

Supplement: Supplementary file 2 [file Data_Sheet_3_v1.pdf]

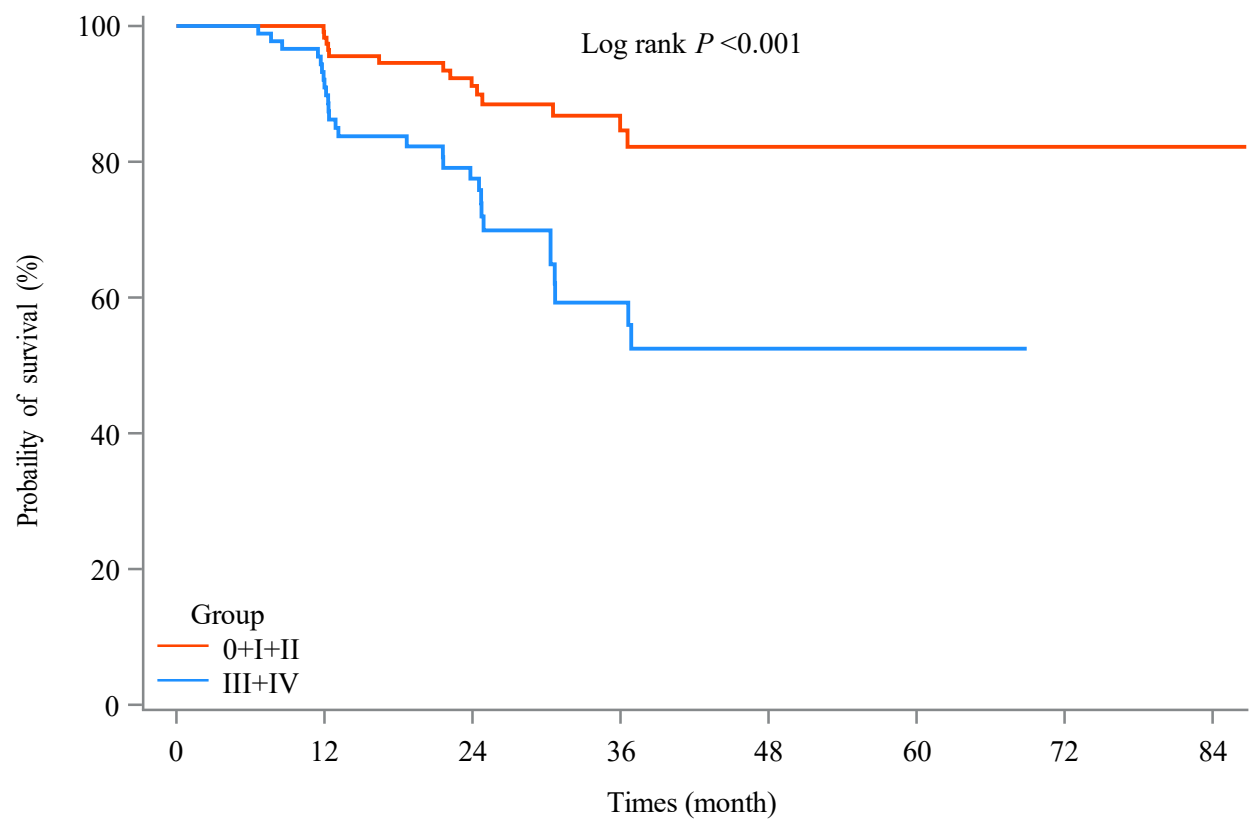

|        |             |     |    |    |    |   |   |   |
|--------|-------------|-----|----|----|----|---|---|---|
|        | No. at risk |     |    |    |    |   |   |   |
| 0+I+II | 125         | 114 | 80 | 39 | 28 | 8 | 2 | 1 |
| III+IV | 94          | 81  | 49 | 18 | 11 | 2 | 0 |   |

Supplement: Supplementary file 3 [file Data_Sheet_4_v1.pdf]

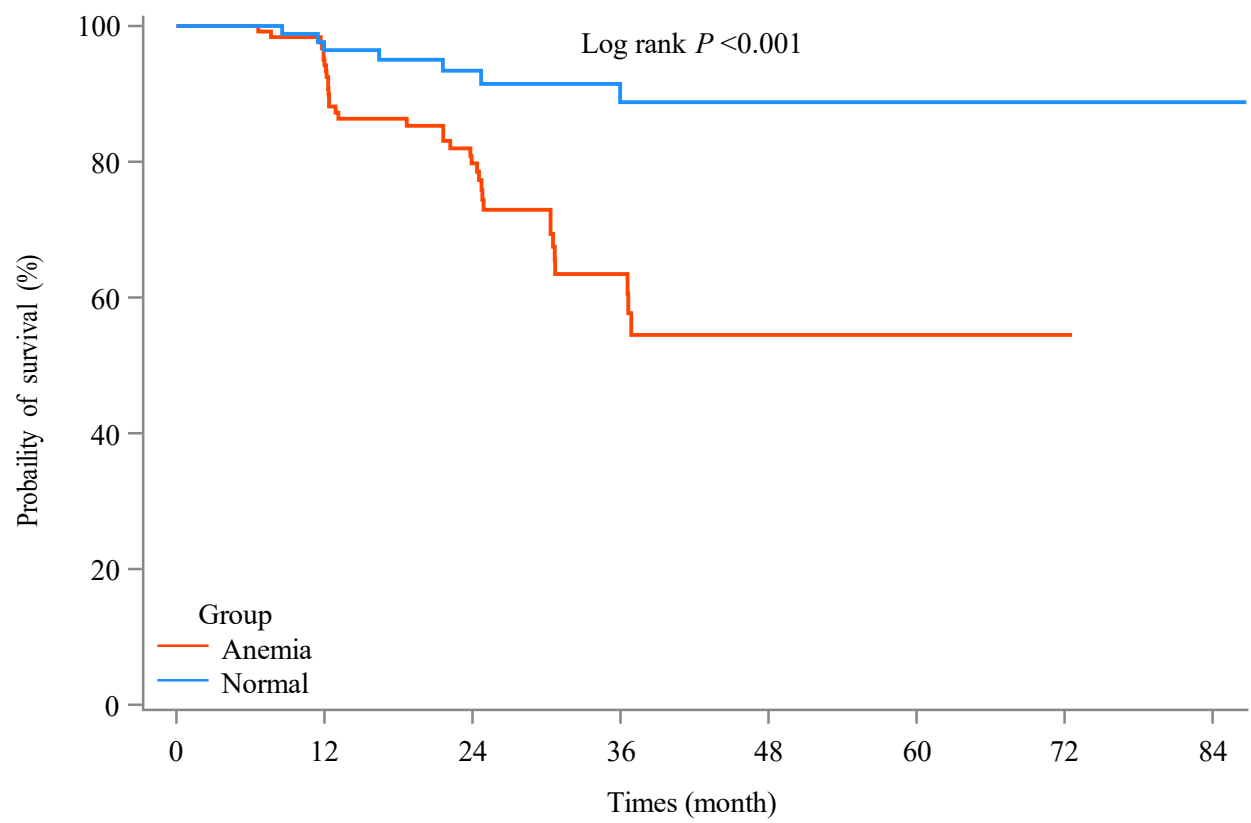

|        | No. at risk |     |    |    |    |   |   |   |
|--------|-------------|-----|----|----|----|---|---|---|
| Anemia | 126         | 114 | 72 | 24 | 14 | 3 | 1 | 0 |
| Normal | 93          | 81  | 57 | 33 | 25 | 7 | 1 | 1 |

Supplement: Supplementary file 4 [file Data_Sheet_5_v1.pdf]

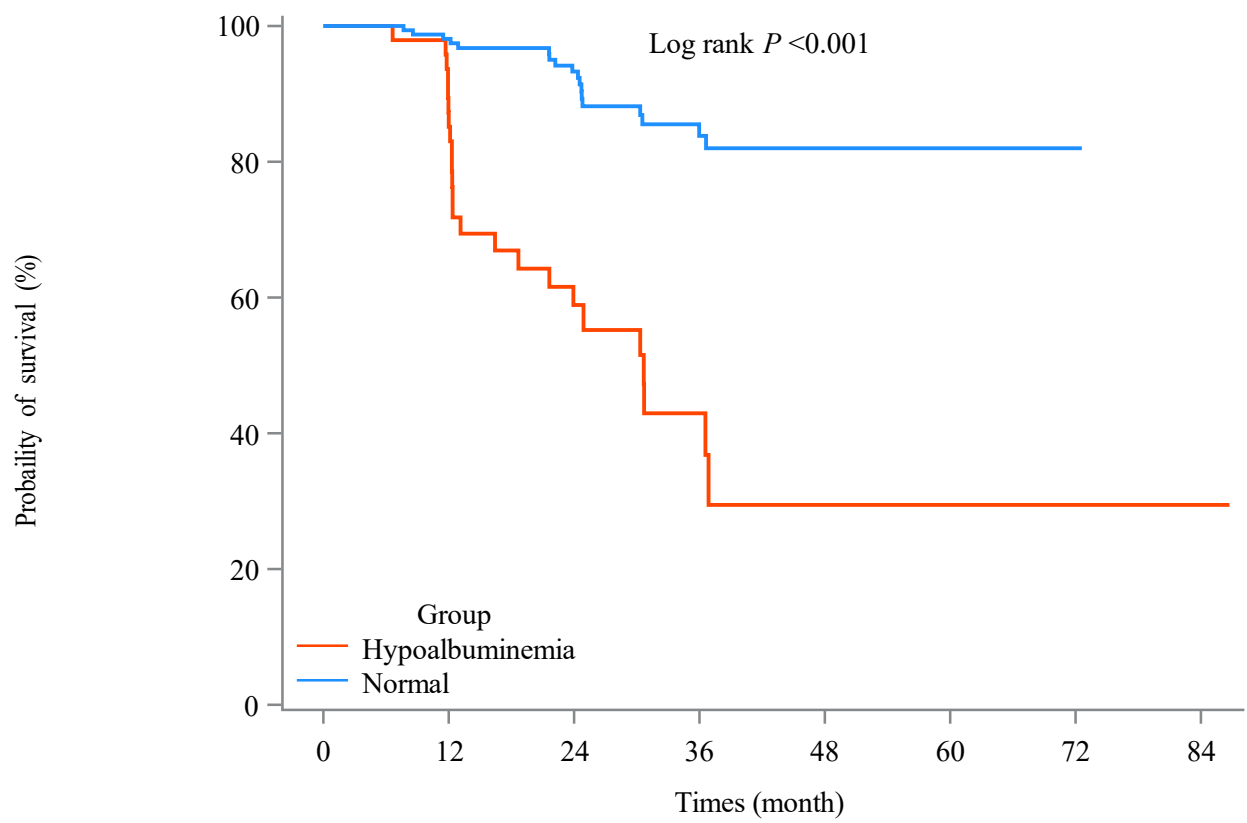

|                 |             |     |     |    |    |   |   |   |
|-----------------|-------------|-----|-----|----|----|---|---|---|
|                 | No. at risk |     |     |    |    |   |   |   |
| Hypoalbuminemia | 49          | 41  | 22  | 8  | 3  | 1 | 1 | 1 |
| Normal          | 170         | 154 | 107 | 49 | 36 | 9 | 1 | 0 |

Supplement: Supplementary file 5 [file Data_Sheet_6_v1.pdf]
